# Prolonged Response Induced by Single Agent Vemurafenib in a *BRAF V600E* Spinal Ganglioglioma: A Case Report and Review of the Literature

**DOI:** 10.3389/fonc.2019.00177

**Published:** 2019-03-26

**Authors:** Louis Garnier, François Ducray, Clotilde Verlut, Marcella-Ionela Mihai, Françoise Cattin, Antoine Petit, Elsa Curtit

**Affiliations:** ^1^Department of Medical Oncology, University Hospital of Besançon, Besançon, France; ^2^Department of Neuro-Oncology, Hospices Civils de Lyon, Lyon, France; ^3^Department of Neurology, University Hospital of Besançon, Besançon, France; ^4^Department of Pathology, University Hospital of Besançon, Besançon, France; ^5^Department of Radiology, University Hospital of Besançon, Besançon, France; ^6^Department of Neurosurgery, University Hospital of Besançon, Besançon, France

**Keywords:** central nervous system tumor, spinal cord, ganglioglioma, *BRAF*, vemurafenib, safety, tumor regression

## Abstract

Spinal ganglioglioma is a rare low-grade, slow-growing tumor of the central nervous system affecting mostly children and young adults. After surgery, some patients show tumor recurrence and/or malignant transformation. Gangliogliomas harbor molecular deficiencies such as mutations in the B-rapidly accelerated fibrosarcoma (*BRAF*) gene, resulting in activation of a downstream signaling pathway and cancer development. Vemurafenib is a *BRAF* inhibitor used to treat patients with *BRAF V600E*-mutated cancer. Although a few studies have reported the clinical responses in gangliogliomas, the sequence and duration of treatment have not been established. We describe a case of an adult with a progressive *BRAF V600E* mutant spinal cord ganglioglioma 9 years after surgery who was treated with vemurafenib. This treatment resulted in a partial response within 2 months, which was sustained for more than a year. The patient then decided to stop treatment because of side effects. Despite this decision, the tumor showed no sign of progression 21 months after treatment discontinuation. This is the first reported case of a response to vemurafenib in an adult with progressive spinal cord *BRAF V600E*-mutated ganglioglioma which was sustained after treatment discontinuation.

## Introduction

Ganglioglioma is a neuronal tumor representing 1% of all primary brain tumors and nearly 5% of pediatric and young adult central nervous system tumors. Histologically, ganglioglioma is composed of both neoplastic neuronal cells and neoplastic glial cells. The glial cells contingent includes astrocyte cells with atypia (1). Most (>90%) gangliogliomas are classified as grade I according to the 2016 World Health Organization (WHO) classification and are genetically defined by multiple alterations (2). Ganglioglioma are typically located in the brain, most often in the temporal lobe and rarely in the spinal cord (3). The cornerstone of curative treatment for ganglioglioma is total surgical resection. The prognosis depends on the quality of surgery (4–9). Complete resection is not always possible, frequently because of the proximity of eloquent structures or vessels. Moreover, even after imaging-confirmed complete resection, recurrence can occur (10).

Treatment strategies are limited for recurrent disease. Radiotherapy has been indicated for high-grade or incompletely resected low-grade ganglioglioma, but these recommendations are not based on high scientific levels of evidence (4, 5, 11–15). Some cases of malignant transformation after radiotherapy have been reported (16, 17). Chemotherapy and systemic therapy are not recommended in the clinical routine and can be discussed on a case-by-case basis after the failure of local therapies (5, 15).

*BRAF* is located on chromosome 7 (7q34) and encodes B-raf, a serine/threonine protein kinase that mediates the cellular response to growth signals (18). B-raf is a member of the Ras/Raf/MEK/ERK/MAP kinase pathway, which is frequently activated in human cancers. More than 30 mutations have been detected in *BRAF*. One of the mutational hot spots of *BRAF* is at nucleotide 1799; mutations at this site lead to the exchange of valine with glutamate at amino acid position 600. The *BRAF V600E* mutant constitutively activates downstream signaling pathways. The *BRAF V600E* mutation occurs in 10–60% of gangliogliomas depending on the study and anatomic site, with lower frequency in the spinal cord (2, 19–21). This mutation appears to be associated with lower recurrence-free survival (22). Therefore, *MAPK* pathway inhibition is an attractive treatment option for recurrent or high-grade ganglioglioma (23).

Vemurafenib is a competitive small-molecule serine–threonine kinase inhibitor that functions by binding to the ATP-binding domain of mutant *BRAF*. Vemurafenib was first licensed for the treatment of advanced melanoma (24). Its administration is now known to be safe and effective for melanoma brain metastases and can result in tumor regression (25). Some case reports have shown an objective tumor response to *BRAF* inhibitor treatment alone or in combination with chemotherapy or targeted therapy in pediatric and young adult *BRAF V600E* gangliogliomas (26–37). However, there are no reports of a prolonged response to monotherapy with vemurafenib in an adult with progressive grade I ganglioglioma. There is a lack of data regarding the use of vemurafenib in gangliogliomas. Particularly, it is unknown how long this treatment should be administered in responding patients. Herein, we describe a case of successful treatment with vemurafenib in a patient with a *BRAF V600E*-mutated progressive cervical spinal cord ganglioglioma, with a stable disease 21 months after treatment discontinuation.

## Case Report Presentation

### Clinical History and Histological Findings

**Figure 1:**
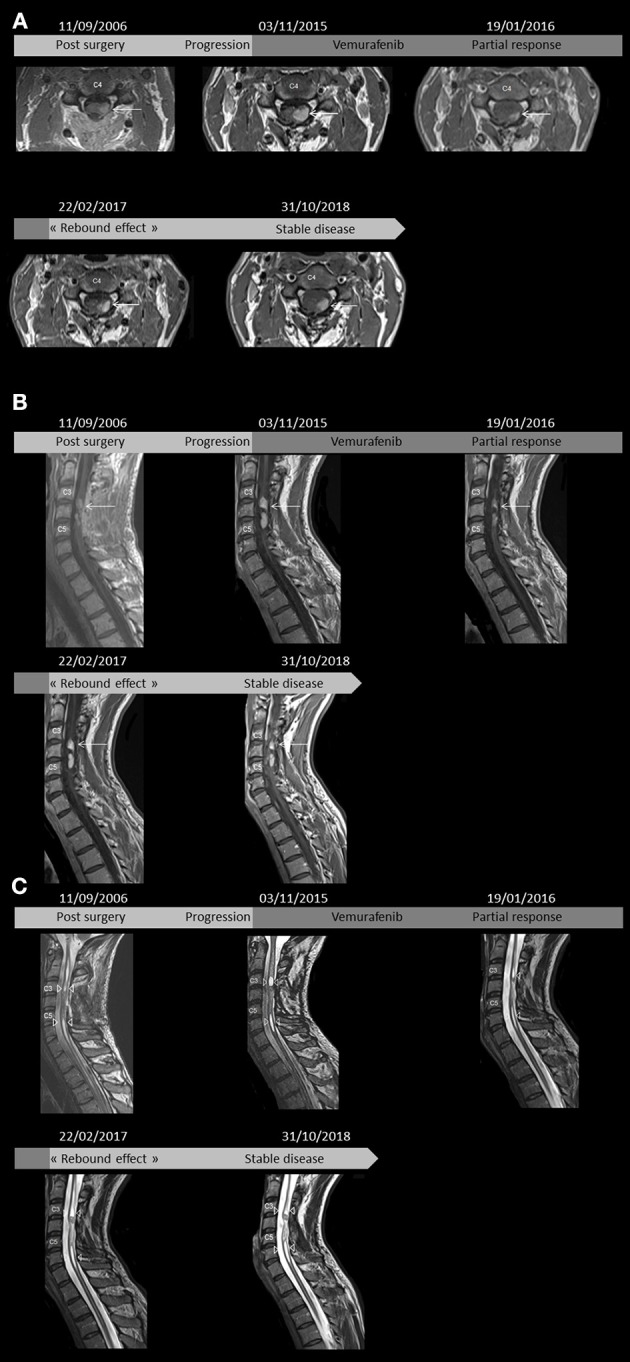
Axial T1-weighted images between C3 and C4 of T1-weighted cervical spine magnetic resonance imaging (MRI) after gadolinium injection administration show dominant, patchy intense enhancing left-sided lesion within the spinal cord (arrows) 1 month after surgery, in pre-treatment with vemurafenib, 8 weeks after the beginning of vemurafenib, 2 months after vemurafenib discontinuation, and last follow-up **(A)**. Sagittal post contrast T1-weighted images revealed lesions between C3 and C4 as well as C4 and C5 (arrows) 1 month after surgery, in pre-treatment, 8 weeks after beginning treatment, 2 months after discontinuation, and last follow-up **(B)**. Sagittal T2-weighted images showing syringomyelia (arrowheads) rostral and caudal to the intramedullary tumor 1 month after surgery, at pre-treatment, 8 weeks after beginning treatment, 2 months after discontinuation, and last follow-up. Note the T2 hyperintensity in the spinal cord above and below the syringomyelia without associated enhancement **(C)**.

A 22-year-old male referred to the emergency department in July 2006 for fluctuating paresthesia with motor dysfunction of the left arm and leg associated with cervical pain, which had been evolving for 1 year. Otherwise, his medical clinical history was unremarkable. Magnetic resonance imaging (MRI) of the spine revealed a suspicious lesion within the left spinal cord at the levels of C3–C5. The patient underwent subtotal resection in August 2006. An MRI of the spine 1 month following surgery showed a residual tumor of 27 × 8 mm with strong patchy enhancement following gadolinium administration within the left spinal cord at the level of the bottom of C3 to the top of C5, isointense T1 signal, and heterogeneously hyperintense T2 signal. There was an associated syringomyelia at the rostral and caudal aspects of the enhancing tumor, mostly from C2 to C7. Moreover, T2 hyperintensity was observed in the spinal cord above and below the syringomyelia without associated enhancement (Figure 1).

**Figure 2:**
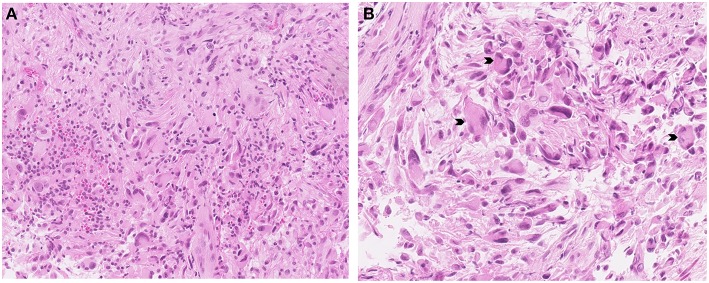
Astrocytic glial cell population with grouped ganglion cells, marked in hematoxylin-Eosin-Saffron staining (original magnification X18) **(A)**, neuronal components characterized by larger numbers of binucleated or multinucleated cells (arrowheads) with a cellular glial background evaluated by hematoxylin-Eosin-Saffron staining (original magnification X28) **(B)**.

The histological examination of the lesion showed a tissue with mixed glial and neuronal components (Figures 2A,B), the presence of fusiform cells with anisonucleosis, sustained by blood vessels with thickened wall surrounded by lymphocytic cuffs, with eosinophilic granular bodies, and Rosenthal fibers. Binucleated neurons were visualized by calretinine, neurofilament, and synaptophysin staining. Many glial cells showed S100 and CD34 immunoreactivity and diffuse glial fibrillary acidic protein. The Ki-67 labeling index was very low (<1%) and some parts of the tissue were positive for P53 in immunohistochemistry analysis. Molecular analysis revealed immunoreactivity to isocitrate deshydrogenase gene 1 (*IDH1 R132H*) and a loss of chromosome 9p. Despite the presence of an *IDH* mutation, central pathological review led to the diagnosis of WHO grade I ganglioglioma (1).

Postoperatively, the patient maintained his neurological symptoms and had Brown-Sequard syndrome and micturition dysfunctions.

The patient was followed up with for 9 years until MRI demonstrated tumor progression. Tumor measurements were then 36 × 12 mm, corresponding to a 50% increase in size (Figure 1). At this time, a second resection was considered too risky and gross tumor resection was not possible. No other treatment was administered because of the lack of proof of chemotherapy and radiotherapy interest. This was consistent with increased arm and leg dysfunction.

### Gene Testing

Molecular testing for evaluation of target therapy was implemented using tissue collected during surgery after obtaining written informed and signed consent. In July 2015, genomic DNA was extracted from the tumor tissue with a QIAamp® DNA mini Kit (QIAGEN, Hilden, Germany) for standard direct sequencing of exon 15 of *BRAF*, which was analyzed by using a SNaPshot® kit (Thermo Fisher Scientific, Waltham, MA, USA). The results revealed a *V600E BRAF* mutation and no mutation in *RAS*.

### Patient Management and Outcomes

**Figure 3:**
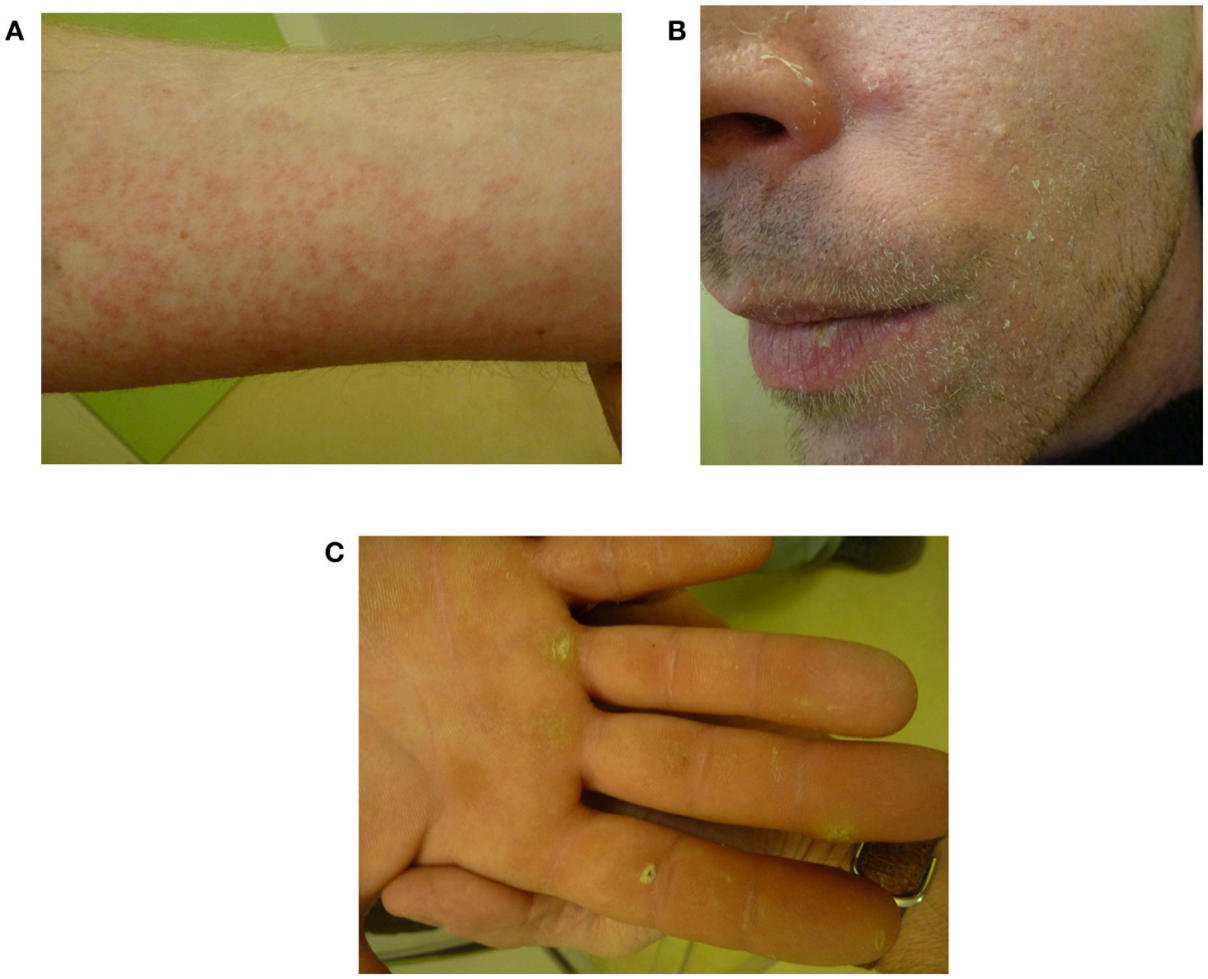
Dermatological toxicities after 13 months of vemurafenib, representing a grade II maculopapular rash **(A)**, microcysts **(B)**, and hyperkeratosis as part of palmar-plantar erythrodysesthesia syndrome **(C)**.

Based on these results, in November 2015, the patient was started on vemurafenib 960 mg orally twice daily [100% of the recommended dose in adults for melanoma (24)]. This treatment was determined as part of “AcSé,” a French program known as “Secure access to innovative targeted therapies” (38). After 8 weeks of treatment, the patient was neurologically stable and brain MRI showed a >50% decrease in tumor size (Figure 1). A steady partial response was observed for more than 13 months. Toxicities were measured by the Common Terminology Criteria for Adverse Events v4.0 and included grade I myalgia, arthralgia, and asthenia as well as grade I maculopapular rash (folliculitis with microcysts on legs and arms treated with topical retinoids). After 13 months of treatment, the patient decided to stop the treatment because of grade II photosensibility and other dermatological side effects (Figures 3A–C). To manage his rashes, folliculitis, and microcysts, the patient applied glycerol as a topical emollient, 30% pure urea cream, and Trétinoine (topical retinoid). His palmar-plantar erythrodysesthesia syndrome (hyperkeratosis) was treated with topical fluorouracil/salicylic acid and even curettage for some areas. Photoprotection was achieved by applying sun cream during treatment. No topical steroid was used. His Eastern Cooperative Oncology Group Performance Status decreased to 2 because of grade II asthenia. Two months after stopping treatment, MRI revealed that the disease was stable and had not significantly progressed according to RANO criteria (39) (Figure 1). Six months after stopping vemurafenib, grade I dermatological side effects persisted but the patient had recovered to a normal Performance Status and MRI showed no signs of progression.

Twenty-one months after vemurafenib discontinuation in October 2018, MRI and neurological examination showed stable disease (Figure 1) and the patient had no side effects. Although the disease is incurable nature, his neurologic and cognitive functions and quality of life were preserved.

## Discussion

To our knowledge, this is the first case of an adult with progressive *BRAF V600E-*mutated spinal ganglioglioma successfully treated with vemurafenib as a single agent and with ongoing and prolonged stable residual disease 21 months after vemurafenib discontinuation.

### Epidemiology

The first description of ganglioglioma was detected in 1870 by Loretz and further studied in 1926 by Perkins. Ganglioglioma is a rare tumor of the central nervous system accounting for 1–1.5% of all spinal tumors (4, 40, 41). Gross total resection is the most reliable treatment (10, 42). While the larger part of this disease occurs in the temporal lobe, causing epilepsy (5) and showing a male preference, its spinal location makes treatment difficult, increasing the risk of recurrence (10, 42). Dudley et al. used the large Surveillance Epidemiology and End Results database, which represents nearly one-third of North America's population, and identified 348 children with low-grade gangliogliomas to study their characteristics (8). This was the largest study to evaluate the spinal location of this rare tumor. Spinal cord gangliogliomas represented 3.5% of cases, with nearly 100% of survival at 5 years and the highest percentage of gross total resection of more than 83%.

### Imaging Findings

MRI findings for supratentorial ganglioglioma can be divided into three groups: cystic, cystic-solid and solid (43). For intramedullary ganglioglioma, imaging manifestation varied considerably (44). In a recent study of 142 cases, all gangliogliomas in the cervicomedullary junction and all *BRAF* mutation-positive ganglioglioma were contrast-positive (21). Our case had a solid lesion with patchy enhancement and a cystic component, which was consistent with previous reports (45). Furthermore, the rapid but not significant regrowth of the tumor after treatment discontinuation in our case may be associated with a “rebound effect,” as described previously (30, 37). This is analogous to pseudo-progression. Indeed, pseudo-progression is commonly observed in asymptomatic patients and occurs at weeks and up to 3 months after treatment. However, previous studies showed that pseudo-progression occurs because of radiotherapy and is characterized by transient T1 gadolinium enhancement resulting from breakdown of the blood brain barrier, which typically resolves spontaneously without treatment (46). Pseudo-progression has also been described in patients treated with immunotherapy, but its incidence is unknown because of the lack of available data. In the two previous reports (30, 37), the therapeutic benefit was again achieved after vemurafenib re-introduction. Re-activation of the Ras/Raf/MEK/ERK/MAP kinase pathway may occur, but the biological mechanism remains unclear.

### Outcomes and Treatments

In a retrospective review of 58 patients (median age at diagnosis of 8.5 years) who underwent surgical resection, the 5- and 10-year overall survival rates were 89 and 83%, respectively. The spinal cord location was associated with a 3.5-fold increased risk of recurrence compared to cerebral gangliogliomas (47).

The efficacy of chemotherapy for adjuvant or recurrent ganglioglioma is uncertain and remains controversial (48), with a high risk of serious adverse events. Recommendations for the use of radiotherapy at progression are based on case reports and small cohorts, particularly in the spinal cord (49). Radiotherapy may result in a better local control for subtotal resection in the supratentorial location, but does not improve overall survival (4, 5, 11–15). Some case reports even suggested that radiotherapy can result in malignant transformation (16, 17). Based on these reports, we did not treat our patient with radiotherapy or chemotherapy.

### Histopathological and Molecular Features

The presence of the *BRAF V600E* mutation suggests that use of *BRAF* inhibitors are efficient for treating recurrent gangliogliomas. *BRAF* mutation appears in 8% of human cancers (50). The *BRAF V600E* mutation was found more often in pediatric low-grade than in high-grade gliomas (2, 18, 51), likely because low-grade gliomas are the most frequent brain tumors in children (52). Patients with *BRAF V600E* mutation exhibit shorter progression-free survival (22, 53). However, the prognostic value of this mutation in recent studies is controversial (54). Thereby, Jones et al. suggested that caution should be used when interpreting the *BRAF* mutations status as an independent prognostic marker (55).

*BRAF V600E* mutations were detected in nearly 20% of gangliogliomas in a screen of 1,320 nervous system tumors (19). This was the second most frequently *BRAF*-mutated cerebral tumor entity after pleomorphic xanthoastrocytoma. In another cohort, 50% gangliogliomas were mutated (20). In a recent series, *BRAF V600E* mutations were detected in 38% of cases but all spinal cord gangliogliomas were wild-type (56). Another group identified only two tumors among 19 (10%) intramedullary gangliogliomas harboring a *BRAF V600E* mutation (3).

Young adult age, synaptophysin positive tumor, lymphocytic cuffs, and a high Ki67 level (mean 2.5%) have been shown to be associated with the *BRAF V600E* mutated status (57). However, the results of Ki67 analysis did not reach statistical significance. Moreover, the mutation appears to be present in the neuronal component or both the neuronal and glial components, but never in the glial component alone (5, 57–59). *IDH* mutations were reported in 8% of cases in a series of 100 gangliogliomas (60). The presence of this mutation was correlated with a greater risk of recurrence and malignant transformation. In the 2016 WHO classification, detection of *IDH1* mutation in a tumor resembling a ganglioglioma strongly supported the diagnosis of an infiltrating glioma with ensnared neurons (1, 61). However, as observed in our patient, it has been increasingly recognized that some circumscribed gliomas can harbor mutations typically encountered in diffuse gliomas (such as *IDH* and histone mutations) (62–65). Occasionally, H3K27M mutations have been reported in midline gangliogliomas (66). In contrast to diffuse gliomas, H3K27M mutations do not appear to be associated with a poor prognosis in circumscribed gliomas. The H3K27M mutation status was not determined in our case.

### Ganglioglioma Treated With *BRAF* Inhibitors: Review of Case Reports

There are some previous descriptions of the efficacy of vemurafenib and dabrafenib (another *BRAF* inhibitor) in low- and high-grade gliomas other than ganglioglioma (35, 67–71). In a basket study with vemurafenib in *BRAF V600E* mutation-positive non-melanoma cancers (35), the objective response rate in *BRAF*-mutant gliomas was 25%. Previously reported cases of gangliogliomas treated with a *BRAF* inhibitor are listed in Table 1 (26–37). The response to vemurafenib in our patient was consistent with the response to *BRAF* inhibitors observed in previously reported cases, including one case of spinal ganglioglioma in a 2-year-old child (29). However, all cases except for one were located in the cerebrum or were brainstem gangliogliomas, with half of the cases being anaplastic gangliogliomas (9/19) (28, 32–36). In eight cases (8/19) (26, 27, 32–34, 36, 37), the *BRAF* inhibitor was associated with another treatment or surgery, making the analysis of the response to the *BRAF* inhibitor difficult. Based on the analysis of the present case and previously reported cases, a complete response was obtained in 15% (3/20) and partial response in 50% (10/20) of cases at a median of 3.2 months after starting treatment and the estimated progression-free survival was 14 months. In 12 patients in whom a *BRAF* inhibitor was administered as a single agent, the response rate was 50% (6/12) (one complete response and partial response in all other patients). Additionally, 33% (4/12) showed stable disease and 17% (2/12) showed progressive disease. The estimated progression-free survival was 11 months. The median follow-up time after starting treatment was 14.5 months, while this time period was 36 months in our case, including 21 months of stable disease after discontinuation. The present case is remarkable because our patient had spinal ganglioglioma treated with vemurafenib alone and a long follow-up. Interestingly, 2 months after vemurafenib disruption for patient convenience, a moderate (<25%) increase in the size of the contrast enhancement was observed, after which the tumor remained stable in subsequent MRIs. This rapid but not significant regrowth was consistent with the previous report of a “rebound effect” following vemurafenib disruption after protracted exposure to this treatment (30). In this situation, vemurafenib re-challenge may be effective (30), but the present case suggests that close follow-up is another option, as further tumor progression may not systematically occur. In recent years, *BRAF/MEK* double blockade with vemurafenib and cobimetinib or dabrafenib and trametinib was shown to be a more effective strategy than targeting *BRAF* alone in patients with *BRAF*-mutant advanced melanoma (72). Dual *BRAF/MEK* inhibition has also been suggested as a promising activity in BRAF-mutant gliomas that may overcome (36, 73) vemurafenib resistance. A prospective study is needed to assess the efficacy of this combination in gangliogliomas.

**Table 1:** Comprehensive list of reported cases of ganglioglioma treated with BRAF inhibitor with reported outcomes.

**References**	**Age (year)/sex**	**Location**	**Symptoms/signs (clinical features)**	**MRI**	**Surgery**	**Treatment before BRAFi**	**Time to recurrence**	**BRAFi**	**Concomitant treatment to BRAFi**	**Side effects**	**Dose modification**	**Response**	**Follow up/recurrence post BRAFi**
Rush et al. (26)	13/F	Brainstem (cervicomedullary)	Paraparesis	Enhancement	Partial	Proton RT (PO)	14 months	V	Vinblastine	Arthalgias, keratosis, telangiectasia	Yes (side effects)	PR at 6 weeks	3 months/None
Shih et al. (27)	21/M	Temporal lobe and posterior brainstem	Headaches, gait disturbance	Enhancement	GTR	Vincristin + Carboplatin (PO)/ RT + TMZ/IRI + BVZ	11 years	D	Gemfibrozil	None	None	PR at 2 months	3 months/ Yes
Bautista et al. (28)	2 children/NM	Thalamus (*n* = 1) Peduncul (*n* = 1)	NM (*n* = 2)	NM(*n* = 2)	Debulking(*n* = 2)	IRI + BVZ then RT + TMZ (*n* = 1)/4 CT (*n* = 1)	1 month (*n* = 1) on therapy (*n* = 1)	V (*n* = 2)	Surgery (*n* = 1)	Hepatotoxicity, skin photo toxicity (grade 1 and 2)	Yes (side effects) (*n* = 2)	SD (*n* = 1) PR at 4 months (*n* = 1)	4 and 20 months/Yes (*n* = 1)^*^ None (*n* = 1)
Del Bufalo et al. (29, 37)	2/M	Cervical spinal cord to C5	Respiratory insufficiency	Cystic component, syringomyelia	Debulking	CT (PO) then surgery	3 months	V	RT at 24 months after the start	Skin rash (grade 3)	None	PR at 3 months	54 months/Yes^**^
Del Bufalo et al. (37)	3 children/NM	Vermis (*n* = 1) M.oblongata (*n* = 1) Midbrain (*n* = 1)	NM (*n* = 3)	NM (*n* = 3)	Partial (*n* = 3)	None (*n* = 3)	NM	V	None	Skin rash (grade1) (*n* = 2)	None (but 480 mg/day)	CR at 6 months (*n* = 1) SD (*n* = 1) NM (*n* = 1)	2,13 and 40 months/None (*n* = 2)
Aguilera et al. (30)	8/M	Brainstem	Sensory disturbance	Enhancement	STR	Proton RT (PO) then surgery	6 months	V	None	Hypoalbuminemia, pruritus (grade 1), maculopapular rash (grade 2)	None	PR at 6 months	12 months/None ^***^
Chamberlain (31)	3 adults/NM	Frontal lobe (*n* = 2) Temporal lobe (*n* = 1)	NM	NM	STR (*n* = 1)GTR (*n* = 2)	RT (*n* = 3) then TMZ (*n* = 3)	NM (*n* = 3)	D	None	NM (*n* = 3)	None	SD (*n* = 2) PR (*n* = 1)	PFS 4, 7, 10 months
Meletath et al. (32)	11/M	Parietal lobe	Hemiparesis, aphasia	Enhancement	STR then partial 9 years later	RT + TMZ (PO)	4 months	D	TTFields	Febrile reaction	Yes (side effects)	CR at 2 months	2 years/None
Marks et al. (33)	16/F	Temporal lobe	seizure	Enhancement, cystic component	STR	RT + TMZ (PO)	1 year	V then D	D + Trametinib	Allergic reaction (grade 4) to V	None	CR at 8 weeks	6 months/None
Beland et al. (34)	51/F	Temporal lobe	Brain hemorrhage	NM	Partial	None (progression post op)	PO	D	D + Trametinib + RT	Nausea, blurred vision, peripheral edema	None	PR at 3 months	8 months/None
Kaley et al. (35)	3 NM	Cerebral not specified (*n* = 3)	NM	NM	NM	RT (*n* = 3) TMZ (*n* = 2)	NM	V	None	NM	NM	PR at 4months (*n* = 1) PD at 2 months (*n* = 1) NM (*n* = 1)	7.5 months/yes
Touat et al. (36)	28/M	Temporal lobe	NM	Enhancement	Partial	RT	27 weeks	V then V + MEKi	V + Cobimetinib	Photosensitivity	NM	PR at 4 months with V and CR at 3 months	14 months/yes then16 months

* reintroduction = PR 1 months then died 2 months

** progression 24 months after the start of the treatment treated with RT

*** *reintroduction 3 months after stopping = PR 14 months*.

*A, anaplastic ganglioglioma; NM, not mentioned; A, anaplastic; PO, post-operative; RT, radiotherapy; TMZ, temozolomide; IRI, irinotecan; BVZ, bevacizumab; V, vemurafenib; D, dabrafenib; CT, chemotherapy; PR, partial response; STR, subtotal resection; GTR, gross total resection; SD, stable disease; PD, progression disease; PFS, progression free survival*.

In our case, the patient asked for treatment discontinuation because of dermatological toxicity. To avoid treatment discontinuation, intermittent dosing could have been used, which has been shown to result in persistent efficacy and improve tolerability as a means of managing *BRAF* inhibitor toxicity (74). Another possibility may have been switching the patient to another, better-tolerated *BRAF* inhibitor such as dabrafenib or combining the treatment with an *MEK* inhibitor which is paradoxically associated with fewer secondary effects than *BRAF* inhibitors alone (33).

## Conclusion

Treatments after surgery for recurrent or progressive spinal cord *BRAF V600E*-mutated ganglioglioma are scarce and the optimal treatment sequence is unknown. We present a case of a sustained and ongoing response to vemurafenib nearly 2 years after the patient interrupted treatment. In the absence of gold standard management in such cases, the present case suggests that vemurafenib should be considered in *BRAF*-mutant spinal gangliogliomas requiring treatment other than surgery. The *BRAF* mutation should be routinely detected in all gangliogliomas even in cases in which *IDH* mutation suggests diffuse astrocytoma. A safety and pilot efficacy clinical trial of vemurafenib as a single agent against *BRAF V600E* mutant recurrent or refractory low-grade ganglioglioma in children is ongoing (ClinicalTrials.gov Identifier: NCT01748149). Moreover, the association between the *BRAF* and *MEK* inhibitor should be studied in a large cohort, as this treatment may have survival benefits in melanoma (72), and enrollment is currently ongoing for a study of *de novo* low-grade and relapsed or refractory high-grade gliomas (ClinicalTrials.gov Identifier: NCT02684058 and NCT02124772). Second-generation *BRAF* and *MEK* inhibitors are also being evaluated (ClinicalTrials.gov Identifier: NCT02285439 and NCT03429803).

## Ethics Statement

Written informed consent was obtained from the patient for publication of this case report and any accompanying images.

## Author Contributions

LG participated in the treatment of the patient, did the literature search, and drafted the manuscript. EC and CV instructed and participated in the treatment of the patient. EC and FD provided critical revisions of the manuscript for important intellectual content. M-IM carefully reviewed the pathological findings. FC carefully reviewed the radiology findings. AP carefully reviewed the surgical findings. All authors read and approved the final manuscript.

### Conflict of Interest Statement

The authors declare that the research was conducted in the absence of any commercial or financial relationships that could be construed as a potential conflict of interest.
